# A prospective randomized study of the natural history of idiopathic scoliosis versus treatment with the SpineCor brace

**DOI:** 10.1186/1748-7161-7-S1-O24

**Published:** 2012-01-27

**Authors:** C Coillard, A Circo, C Rivard

**Affiliations:** 1 Sainte-Justine Hospital, Montreal, Canada

## Background

The purpose of this randomized study was to evaluate the effectiveness of the Dynamic SpineCor brace [1,2] for early adolescent idiopathic scoliosis (15°-30°) compared to the natural evolution of the disease. 47 patients participated in this study (26 treated and 21 controls).

## Material and methods

The inclusion criteria where: 1) High risk of evolution: family history and/or proven progressive 2) No significant pathological malformation of the spine; 3) Girl or boy; 4) Initial Cobb angle between 15° and 30°; 5) Risser 0, 1 or 2. Assessment of brace effectiveness included; 1) percentage of patients who have 5° or less curve progression and the percentage of patients who have 6° or more progression at skeletal maturity, 2) percentage of patients who have had surgery recommendation/undergone before skeletal maturity.

## Results

At three years follow up a correction was achieved in 50% of treated patient and only in 9.5% of controls, stabilization in 23.1% treated and 33.4% in controls and progression in 26.9 % for the treated group and 59.1% for controls. Three immature patients required surgical fusion while receiving treatment (11.5%) as well as 3 control patients (14.3%). For the control patients we considered as a failure if the Cobb angle worsened by more then 5° from the original angle and the patient then received treatment.

## Conclusions

The SpineCor brace is effective for the treatment of early adolescent idiopathic scoliosis comparing with its natural history. Moreover, the positive outcome appears to be maintained in the long term.
